# Giant conductivity switching of LaAlO_3_/SrTiO_3_ heterointerfaces governed by surface protonation

**DOI:** 10.1038/ncomms10681

**Published:** 2016-02-10

**Authors:** Keith A. Brown, Shu He, Daniel J. Eichelsdoerfer, Mengchen Huang, Ishan Levy, Hyungwoo Lee, Sangwoo Ryu, Patrick Irvin, Jose Mendez-Arroyo, Chang-Beom Eom, Chad A. Mirkin, Jeremy Levy

**Affiliations:** 1 Department of Chemistry and International Institute for Nanotechnology, Northwestern University, Evanston, Illinois 60208, USA; 2 Department of Physics and Astronomy, University of Pittsburgh, 100 Allen Hall, 3941 O'Hara Street, Pittsburgh, Pennsylvania 15260, USA; 3 Pittsburgh Quantum Institute, Pittsburgh, Pennsylvania 15260, USA; 4 Department of Materials Science and Engineering, University of Wisconsin-Madison, Madison, Wisconsin 53706, USA

## Abstract

Complex-oxide interfaces host a diversity of phenomena not present in traditional semiconductor heterostructures. Despite intense interest, many basic questions remain about the mechanisms that give rise to interfacial conductivity and the role of surface chemistry in dictating these properties. Here we demonstrate a fully reversible >4 order of magnitude conductance change at LaAlO_3_/SrTiO_3_ (LAO/STO) interfaces, regulated by LAO surface protonation. Nominally conductive interfaces are rendered insulating by solvent immersion, which deprotonates the hydroxylated LAO surface; interface conductivity is restored by exposure to light, which induces reprotonation via photocatalytic oxidation of adsorbed water. The proposed mechanisms are supported by a coordinated series of electrical measurements, optical/solvent exposures, and X-ray photoelectron spectroscopy. This intimate connection between LAO surface chemistry and LAO/STO interface physics bears far-reaching implications for reconfigurable oxide nanoelectronics and raises the possibility of novel applications in which electronic properties of these materials can be locally tuned using synthetic chemistry.

## Abstract (web summary)

The interface between two oxide materials can play host to numerous exotic phenomenon. Here, the authors observe a four order of magnitude change in the conductance at a lanthanum-aluminate–strontium-titanate interface controlled by surface protonation, which can be reversed by exposure to light.

## Graphical abstract

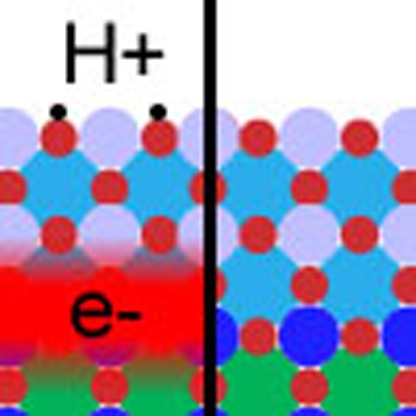

The continued miniaturization of electronics and emergence of novel two-dimensional materials and heterostructures demands that the relationships between electronic properties and surface chemistry be well understood. With traditional (for example, III–V) semiconductor heterostructures, the conducting interface is generally far (>100 nm) from the functionally active surfaces, and consequently surface chemistry plays a minor role. However, for the more recent class of complex oxide heterostructures, that is, the interface between the band insulators SrTiO_3_ (STO) and LaAlO_3_ (LAO), a high-mobility quasi-two-dimensional electron liquid (2DEL)^1^ can form <2 nm or 4 unit cells (uc) from the surface, bringing questions about the role of surface states into sharp focus. Understanding the surface chemistry of these complex-oxide interfaces is key to understanding the array of fascinating properties these materials exhibit, which includes gate-tunable superconductivity^2^,^3^, magnetism^4^,^5^ and strong spin-orbit coupling^6^,^7^. More than a decade after the first reports of conductivity at the LAO/STO interface, the physical mechanism for 2DEL formation has not been fully and convincingly explained^1^,^2^,^8^,^9^,^10^,^11^,^12^,^13^,^14^. Although 2DEL formation is commonly attributed to an electronic reconstruction resulting from band bending in the polar (100) LAO layer, the ‘polar catastrophe' framework does not explain 2DEL formation in (110) LAO/STO samples, which have no polar discontinuity^15^. Furthermore, nominally insulating 3 uc (1.2 nm thick) LAO/STO samples can be rendered locally and persistently conductive via writing with charged scanning probes^10^,^11^. Switchable 2DELs in 3 uc samples implies the presence of a hidden degree of freedom, and initial investigations implicated LAO surface chemistry through the observation that humidity is required to write conductive regions with a scanning probe^16^,^17^. However, the relative importance of surface chemistry and LAO polarity is not well understood and while immersion in solvents has been observed to alter 2DEL mobility^13^, post-synthetic wet chemical conditions that destroy 2DELs in samples with 4 uc or more LAO have not been previously identified. Despite this gap in experimental understanding, theoretical studies have stressed the importance of surface chemistry by linking 2DEL formation to chemical phenomena including the protonation of oxygen in LAO, the generation of oxygen vacancies and adsorption of water^18^,^19^,^20^.

Here we report a large (>4 order of magnitude) reversible switching of the LAO/STO interface conductivity in response to solvent immersion and optical illumination. Through coordinated electrical measurements, X-ray photoelectron spectroscopy (XPS) and solvent immersions, we find that LAO/STO conductivity is correlated with the protonation of the LAO surface. Furthermore, by immersing the samples in various solutions including different pH aqueous solutions, solvents with a range of p*K*_a_, and solutions of molecules that function as proton sponges, we find that a solvent's ability to render the LAO/STO layer insulating is dictated by its ability to deprotonate the LAO surface. Based on these results, we propose a model in which LAO surface protonation dictates the electronic state of the LAO/STO interface. In support of this, we find that this effect diminishes as the LAO film thickness is increased until it effectively disappears in LAO films 7 uc thick. Finally, we find that the conductive state can be directly patterned by illuminating selected regions of the LAO/STO surface, suggesting a future method for realizing reconfigurable electronics based upon LAO/STO interfaces.

## Results

### Conductivity switching by solvent immersion/illumination

**Figure 1:**
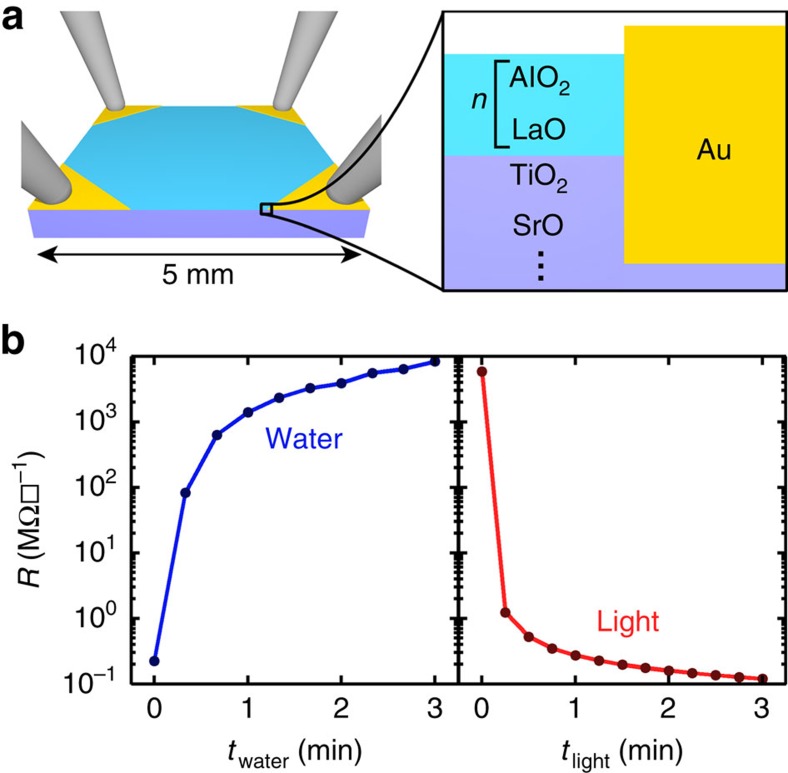
Observation of a reversible transition between a conductive and insulating state by exposure to water and light. (**a**) Schematic showing the bulk electrical transport measurement of 4 unit cell (uc) LaAlO_3_ (LAO) on SrTiO_3_ (STO). (**b**) Sheet resistance *R* as determined by a van der Pauw measurement versus total time exposed to deionized water (*t*_water_, left) and total time exposed to broadband light (*t*_light_, right). Measurement uncertainty is smaller than the data markers in all cases.

Nominally conductive 4 uc LAO films were grown on (100) single-crystal STO substrates using pulsed laser deposition^21^. These samples were subsequently processed (Supplementary Fig. 1) to define four electrodes for Van der Pauw measurements of interfacial sheet resistance *R* (Fig. 1a). To explore the effect of solvent exposure, *R* was measured before and after immersing the sample in deionized water for 20 s and then drying under an N_2_ stream. This immersion-drying-measurement cycle was iterated to generate a plot of *R* versus cumulative immersion time (Fig. 1b—left). Surprisingly, *R* increased by >4 orders of magnitude over the 3 total minutes of immersion. Although this insulating state was stable in the dark under ambient conditions (Supplementary Fig. 2), measurements of *R* following sequential 15 s exposures to broadband light revealed a decrease in *R* commensurate with the solvent-driven increase (Fig. 1b—right). Importantly, owing to the observed light sensitivity, samples were not exposed to light with wavelengths below 520 nm unless otherwise noted.

### XPS studies of LAO surface protonation

**Figure 2:**
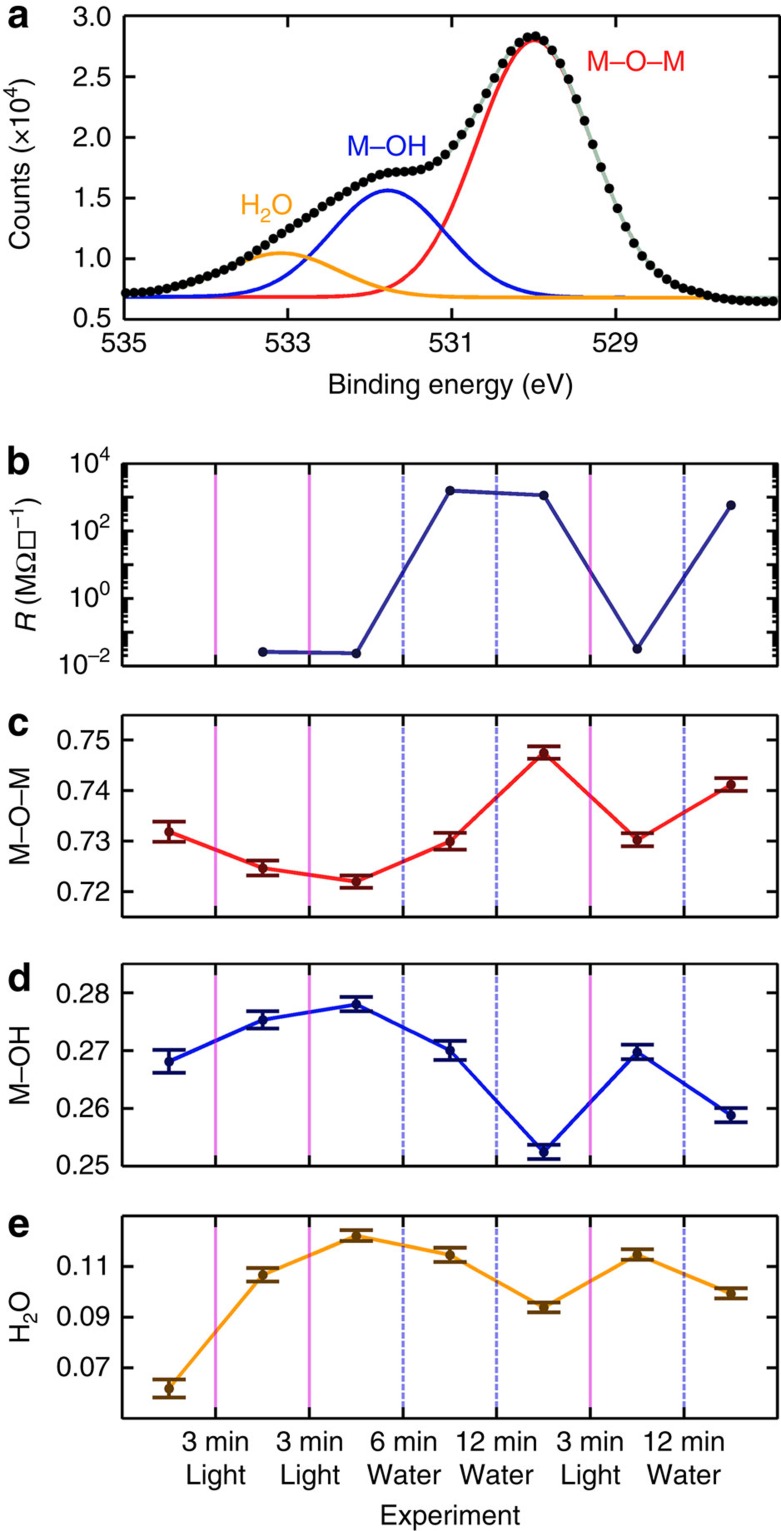
Mechanistic determination through X-ray photoelectron spectroscopy (XPS). (**a**) XPS of 4 uc LAO/STO showing the O1s region. The spectra are fit to the sum of three Gaussians assigned to free water (H_2_O), protonated oxygen (M–OH), and oxygen in the bulk perovskite lattice (M–O–M). (**b**) *R* measured following each treatment, with optical excitation denoted by solid purple lines and water treatment denoted by dashed blue lines. Here uncertainty is smaller than marker size. Evolution of areas of M–O–M (**c**), M–OH (**d**) and H_2_O (**e**) peaks normalized by the sum of M–O–M and M–OH measured while the sample was subjected to optical excitation and water immersion.

To evaluate the chemical changes that correspond to conductivity switching, XPS was employed (Supplementary Fig. 3)^22^. The O1s peak was found to be descriptive of the chemical status of the LAO surface (Fig. 2a), as it exhibited peaks from three discernable species: fully coordinated lattice oxygen (M–O–M), protonated oxygen (M–OH) and oxygen in adsorbed water (H_2_O). These assignments are supported by the literature^23^ and a tilting experiment (Supplementary Fig. 4). A 4-uc LAO sample was subjected to a series of illumination and water treatments while alternatively measuring *R* and chemical composition (Fig. 2b–e). In all cases, exposure to light caused the M–O–M peak area to decrease relative to M–OH, whereas water immersion displayed the opposite effect. Interestingly, the area of the H_2_O peak tracks the M–OH peak, suggesting that the protonated surface binds more strongly to water (Fig. 2e). Although this measurement only provided ratiometric information, the optical absorbance measured by spectroscopic ellipsometry did not appreciably change following illumination or solvent immersion (Supplementary Fig. 5), suggesting that the number of optically active oxygen vacancies is static during these treatments and that the change measured by XPS is due to a change in the protonation state of the LAO surface^24^. It is important to emphasize that optical characterization techniques (for example, XPS and ellipsometry) must be used with caution to study LAO/STO. Specifically, XPS measurements were found to return insulating samples to a conductive state, so it was important to perform XPS after measuring electrical transport. In light of this perturbative effect, XPS should be considered a qualitative measure of surface protonation. Despite these limitations, we consistently observed the trend of protonation increasing with light exposure and decreasing with solvent immersion. We recommend that future studies focus on the use of alternative analytical surface tools such as scanning tunnelling microscopy to provide a more detailed and quantitative picture of the chemical state of the surface.

The combination of XPS and electrical transport measurements suggests that interface conductivity increases with surface protonation. Although this trend is in agreement with first-principles theoretical studies^16^,^18^, immersion in water deprotonating the LAO surface is in apparent contradiction to studies of other perovskite oxides in which water immersion is observed to increase protonation^25^. Thus, an extensive series of experiments were designed to test what properties of solvents drove the conductor to insulator transition.

### Immersion of LAO/STO in solvents

**Figure 3:**
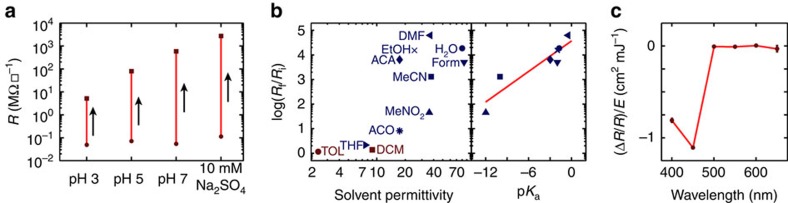
Exploring the mechanism of conductivity switching in LAO/STO heterointerfaces. (**a**) *R* of 4 uc LAO/STO measured before (circles) and after (squares) 1 min of immersion in solvents with varying concentrations of free protons. (**b**) (Left) Log base 10 of the ratio of the resistance *R*_f_ measured following a 2-min immersion in toluene (TOL), tetrahydrofuran (THF), dichloromethane (DCM), acetophenone (ACO), acetaldehyde (ACA), ethanol (EtOH), nitromethane (MeNO_2_), dimethylformamide (DMF), acetonitrile (MeCN), water (H_2_O) or formamide (Form) to the resistance in the conductive state *R*_i_. Blue and red markers represent solvents that do and do not function as Lewis bases, respectively. (right) *R*_f_/*R*_i_ versus the p*K*_a_ of the protonated solvents. (**c**) Ratio of sheet resistance change Δ*R* to *R* normalized by the delivered optical energy *E* following illumination with light of a specified wavelength.

To test the hypothesis that surface protonation dictates interface conductivity, we explored the effect of solvent pH on the conductivity of 4 uc samples (Fig. 3a). Following initialization in a conductive state, *R* was measured before and after immersion in an aqueous solution of sulfuric acid for 1 min and subsequent drying. Interestingly, lowering the pH of the immersion solution from 7 to 3 decreased the magnitude of conductive switching over 100-fold, suggesting that immersion drives an acid–base reaction, where water acts as a Lewis base and removes protons from the surface. This pH range was selected because the isoelectric point of crystalline aluminum oxide is pH∼5 (ref. 26), thus one would expect the surface charge of LAO to vary greatly between pH 3 and 7. To ensure that the effect was not related to the sulfate counterion, the sample was immersed in a sodium sulfate solution that was isomolar with pH<2 sulfuric acid. Immersion in this salt solution increased *R* more than immersion in pH 7 water, indicating that pH is likely determining the observed conductivity change rather than the anionic counterions.

If LAO/STO interface conductivity is dictated by surface protonation, then immersion in solvents besides water that can deprotonate the surface should have an analogous effect. Thus, a series of experiments was performed in which a conductive 4 uc sample was immersed in one of various solvents for 2 min (Fig. 3b—left). Interestingly, only Lewis basic solvents were found to drive conductivity switching; this observation is in support of deprotonation as the mechanism of conductivity switching during solvent immersion. More generally, solvents with relative permittivity *ɛ*≲20 had a small effect, whereas those with *ɛ*>20 substantially changed *R*. This result can be understood by considering that lower permittivity solvents are less able to screen the charge of the protons and overcome the electrostatic attraction between the protons and the electrons in the 2DEL. Importantly, although the resistance increase from immersion in high permittivity solvents did not correlate with solvent permittivity, it correlated extremely well with the p*K*_a_ of the protonated solvent (that is, BH^+^→B+H^+^; Fig. 3b—right), strongly suggesting that the mechanism is dependent upon an acid–base reaction in which the solvent accepts a proton from the surface. In further support of this hypothesis, although nitromethane immersion only increased *R* 30-fold, the addition of 10 mM of a proton sponge to nitromethane resulted in a solution that increased *R* 3,000-fold (Supplementary Fig. 6), indicating that the ability to accept protons is critical for this process. Furthermore, once the sample was prepared in the insulating state, sequential solvent immersion did not appreciably decrease *R* (Supplementary Fig. 7). Care was taken to dry solvents before use, as even the addition of 1% water to tetrahydrofuran was found to triple the effect of immersion (Supplementary Fig. 8).

### Illumination of LAO/STO samples

Although it is plausible that solvent immersion can deprotonate a surface, it is not straightforward that light would reverse this process. In an effort to explore this photoconductivity effect, a 4-uc sample prepared in an insulating state was exposed to monochromatic light of various wavelengths (Fig. 3c). Although *R* did not vary when illuminated by light at wavelengths ≥500 nm, illumination at wavelengths<450 nm decreased *R*. Interestingly, 450 nm photons do not have enough energy to overcome the band gap of either LAO or STO, but oxygen vacancies in STO have optically active transitions between 400 and 460 nm (refs 27, 28), implicating these defect states in the persistent photoconductivity. Following illumination, *R* was found to change less than 10% over 24 h when the sample was kept in vacuum or dry air (Supplementary Fig. 2), but the presence of ambient humidity accelerated the upward drift in *R*, suggesting that water is playing a role in the light-driven process (Supplementary Fig. 2).

**Figure 4:**
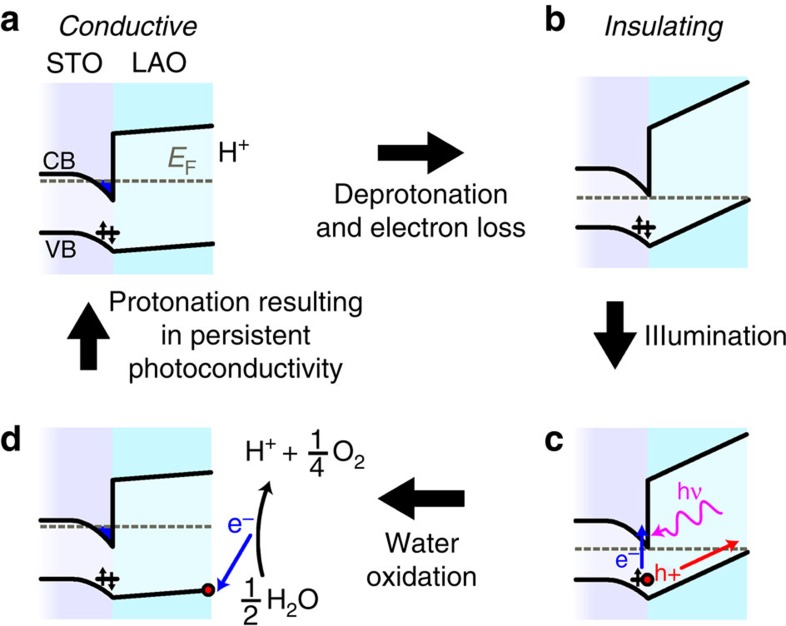
Model of giant conductivity switching in LAO/STO heterointerfaces. (**a**) Initially, the conducting band (CB) and valence band (VB) of the LAO/STO interface bend to create an occupied conductive state at the interface. In addition, a mid-gap oxygen vacancy state in the STO is present below the Fermi energy *E*_F_. (**b**) Solvent immersion removes the proton and causes the electron to leave the interface. (**c**) Ultraviolet light creates electron-hole pairs at the oxygen vacancy, which are separated by the polar LAO potential. Specifically, the hole ‘bubbles up' the VB potential in the LAO and segregates to the surface. (**d**) The surface hole oxidizes adsorbed water, thereby generating a proton. This proton can then interact with Lewis basic sites of the LAO.

Based upon these observations, we postulate a surface protonation-based mechanism for the role of solvent immersion and exposure to light in LAO/STO interfaces (Fig. 4). Specifically, the cycle consists of a chemical change of the protonation state of LAO and an electronic change, in which an electron is exchanged with the environment. Initially, the sample is in the conductive state and the LAO surface is protonated (Fig. 4a). Solvent immersion deprotonates the surface through an acid–base reaction, removing the source of electrostatic attraction that holds electrons at the interface. Thus, an electron leaves the system either through the electrodes or through exchange with the solvent at the leads. With the interface state vacant, the system is insulating (Fig. 4b). Based upon the spectral dependence of the persistent photoconductivity and well-documented presence of oxygen vacancies near the LAO/STO interface^8^, we postulate that exposing the insulating surface to light excites oxygen vacancies at or near the LAO/STO interface. These photogenerated electron-hole pairs are split because the polar potential in the LAO drives the hole towards the LAO surface (Fig. 4c). Once on the surface, the hole is able to accept an electron from an adsorbed water molecule through the oxidative water splitting reaction, 1/2H_2_O→H^+^+1/4O_2_+e^−^ (Fig. 4d), in analogy to the photocatalytic splitting of adsorbed water observed for many perovskite oxides^29^,^30^. Importantly, this process completes both the chemical and electronic cycle by annihilating the hole, thus preventing subsequent electron-hole recombination and creating a proton which is free to associate with Lewis basic sites on the LAO surface (Fig. 4a).

### LAO film thickness

**Figure 5:**
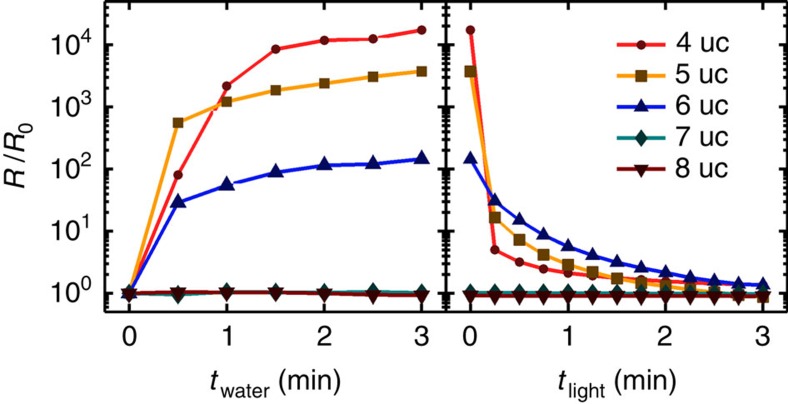
LAO thickness effect on conductivity switching. *R* versus *t*_water_ (left) and *t*_light_ (right) measured for LAO/STO samples with different numbers of unit cells of LAO. Measurement uncertainty is smaller than the data markers in all cases. Note that times *t*_water_ and *t*_light_ indicate cumulative exposure to water and light, respectively. Sheet resistance *R* normalized by the value before the first water immersion *R*_0_=*R*(*t*_water_=0). The data corresponding to the 7 uc and 8 uc samples are difficult to distinguish because neither deviates more than 10% away from unity.

If surface chemistry, rather than band bending, is playing a dominant role in 2DEL formation, then the observed resistivity switching should persist beyond the reported 4 uc critical thickness. To explore this hypothesis, we performed a series of experiments testing the effects of water immersion and illumination on samples with 4, 5, 6, 7 and 8 uc of LAO. Samples were subjected to sequential 30 s immersions in deionized water for a total of 3 min followed by 15 s exposures to light for a total of 3 min (Fig. 5). Interestingly, the 5 uc sample exhibited a three order of magnitude shift in *R* and the 6 uc sample exhibited a two order of magnitude shift in *R,* whereas *R* for both the 7 and 8 uc sample varied under 10% during the entire process. These results support early theoretical work in which the critical thickness was computed to be ∼6 uc and it was hypothesized that the experimentally observed critical thickness of 4 uc was due to defects or surface adsorbates^12^. Although experimental limitations prevented us from observing changes in transport properties faster than the scale of seconds, the fast dynamics of this transition represent a fascinating opportunity for further study.

## Discussion

In apparent contrast to our observations, a prior study has shown that solvent immersion renders LAO/STO heterointerfaces somewhat more conductive (that is, doubling the conductivity)^13^. This discrepancy could be due to two factors: first, in the prior work, it was not stated whether or not the experiments were performed in the dark; however, some experimental variations were attributed to ‘fluctuations in the local illumination.' Second, the study focused on 10 uc LAO/STO, which we expect to have a small variation with solvent immersion (extrapolating from Fig. 5). The only experiment reported on samples with less than 10 uc of LAO utilized acetone, which we expect to have a modest effect both due to the low permittivity of the solvent (∼20ɛ_0_) and the short duration of immersion (10 s). When we replicated these conditions (immersing a conductive 4 uc sample in acetone for 10 s) but kept the sample in the dark, we observed a mere 84% increase in *R*. Thus, we attribute illumination details and choice of immersion conditions to be the cause of the apparent inconsistency with previous work. Our results are consistent with recent coordinated XPS and electrical studies that identify hydrogen species adsorbed from air (i.e. water vapor and hydrogen) as factors that increase the interface conductivity^31^.

It is also worth addressing how this phenomenon could not have been observed previously despite many groups using solvent immersion as part of their fabrication processes. Ultimately, it is easy to expose a sample to enough adventitious light that the conductive state is restored despite a prior solvent immersion. In particular, samples stored in a transparent container overnight in normal workplace illumination conditions would commonly appear fully conductive. Further, temperature-dependent transport measurements of a 4-uc sample with persistent photoconductivity revealed a smoothly varying mobility and carrier density commensurate with previous measurements of LAO/STO samples (Supplementary Fig. 9)^1^. These observations are consistent with the picture that solvent immersion renders the LAO/STO heterointerface insulating and exposure to light recovers the conductivity.

The ability to reversibly switch the conductivity of LAO/STO interfaces through solvent immersion and exposure to light suggests several approaches for realizing large-scale reconfigurable nanoscale patterning of conductive areas. Indeed, preliminary experiments demonstrate that photopatterning can achieve a >2 order of magnitude ratio between the resistance of the patterned and unpatterned regions using standard photolithography (Supplementary Fig. 10). The essential role that surface chemistry plays in mediating 2DEL formation implies that the full diversity of synthetic chemistry may be utilized to design self-assembled monolayers that precisely tune the 2DEL. Taken together, this work provides compelling experimental evidence for surface protonation playing a major role in the conductivity of LAO/STO interfaces and illustrates how this phenomenon can be modulated using solvent immersion and illumination. Although XPS is used here as a qualitative measurement owing to its interaction with the surface, the abundance of protonated oxygen only changed by ∼5%. This naturally raises the question: what change in surface protonation is required to drive the metal to insulator transition? Given that LAO/STO is a system characterized by small changes in parameters (for example, gate voltage, LAO film thickness) producing dramatic effects on electronic transport^9^, it will be fascinating to complement these experiments with quantitative measurements of surface protonation.

## Methods

### Sample preparation

LAO/STO samples were grown via pulsed laser deposition onto (001) STO substrates. Low miscut (<0.10°) single-crystal STO (001) substrates were etched by buffered hydrofluoric acid for 60 s to obtain substrates with a B-site (TiO_2_)-terminated surface. Then, the substrates were annealed in a tube furnace at 1,000 °C for 6 h to make an atomically smooth surface with single-unit-cell height steps. For the growth of epitaxial LAO thin films, a KrF excimer laser (248 nm) beam was focused on a stoichiometric LaAlO_3_ single-crystal target and pulsed at 3 Hz frequency. The growth temperature of substrates was 550 °C and the background oxygen pressure was 10^−3^ mbar. After growing 4 uc of LAO with *in situ* reflection high-energy electron diffraction (RHEED) oscillation monitoring, the samples were slowly cooled down to room temperature. Photolithography, reactive ion etching and electron beam evaporation were subsequently used to introduce four electrodes that contact the heterointerface.

### Measurement of conductivity switching

Solvents (excluding water) were stored for 2 days with molecular sieves to remove trace water and used without further purification. Electrical characterization was performed using a four terminal Van der Pauw measurement with a ±200 mV bias voltage. Illumination was performed using a broadband mercury arc lamp, which produced an optical intensity of 1–2 mW cm^−2^ in the range 400–500 nm. To establish a consistent baseline state, samples were illuminated for 3 min. Solvent treatments consisted of immersing the sample in a scintillation vial with ∼10 ml of the solvent of interest. Following a predetermined amount of time, the sample was removed and dried under a N_2_ stream. XPS measurements were taken following transport measurements and consisted of five high-resolution scans taken at each of three regions of the sample.

## Additional information

**How to cite this article:** Brown, K. A. *et al*. Giant conductivity switching of LaAlO_3_/SrTiO_3_ heterointerfaces governed by surface protonation. *Nat. Commun.* 7:10681 doi: 10.1038/ncomms10681 (2016).

## Supplementary Material

Supplementary Information Supplementary Figures 1-10, Supplementary Methods and Supplementary References.
