# Supplementary material for: Giant conductivity switching of LaAlO3/SrTiO3 heterointerfaces governed by surface protonation
Source: Nat Commun. 2016 Feb 10;7:10681. doi: 10.1038/ncomms10681 (PMC4749969; doi:10.1038/ncomms10681)
Supplement: Supplementary Information — Supplementary Figures 1-10, Supplementary Methods and Supplementary References. [file ncomms10681-s1.pdf]

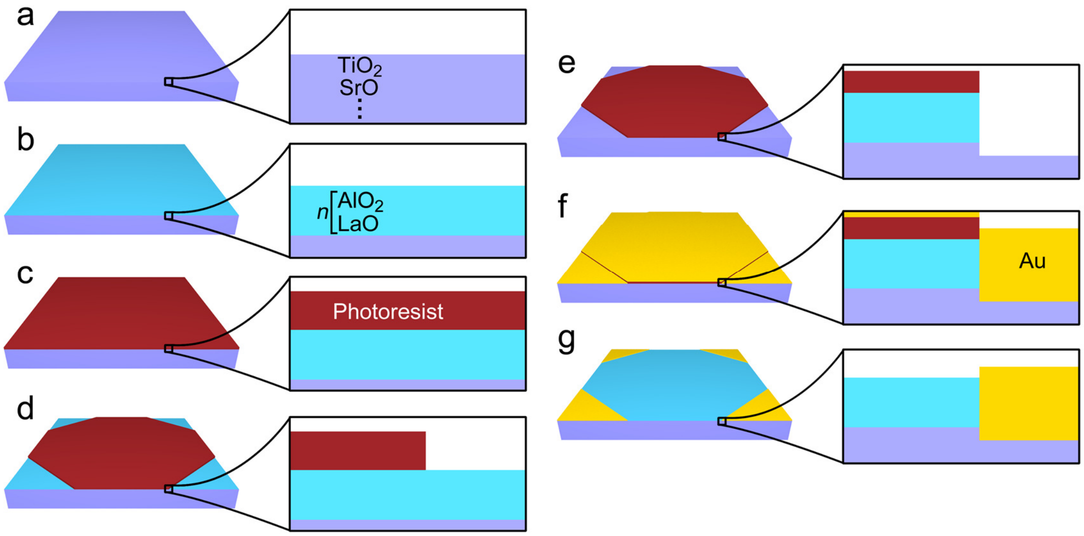

Supplementary Figure 1. Detail of sample preparation. (**a**) (100) $SrTiO_3$ (STO) was prepared for deposition. (**b**) *n* unit cells of $LaAlO_3$ (LAO) were deposited using pulsed laser deposition. (**c**) The sample was spin-coated with photoresist and baked to cure the resist. (**d**) Photolithography and chemical development were used to expose the four corner regions. (**e**) Ar reactive ion etching was used to ablate the exposed LAO surface, exposing the heterointerface. (**f**) Au was deposited using electron-beam evaporation. (**g**) The photoresist was removed by sonication in an acetone bath.

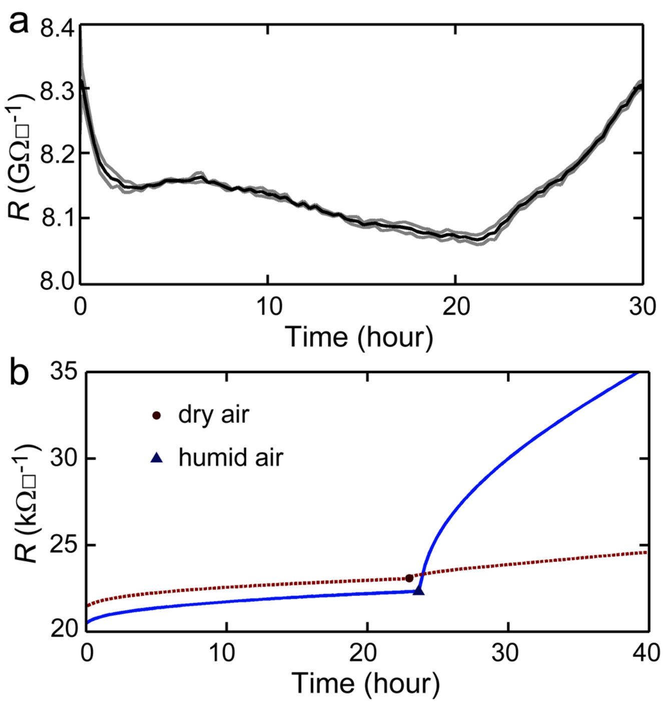

Supplementary Figure 2. Transient response of 4 uc LAO/STO. (**a**) Transient response of sheet resistance *R* following immersion in water for 3 min and being blown dry using N_2. This sample was measured in the dark under ambient atmosphere. The grey lines indicate upper and lower bounds of measurement uncertainty. (**b**) Transient response of *R* following UV exposure in vacuum followed by introduction of dry air (dashed line; circle denotes when air is introduced) and air at 30% relative humidity (solid line; triangle denotes when air is introduced). Measurement uncertainty is smaller than marker size.

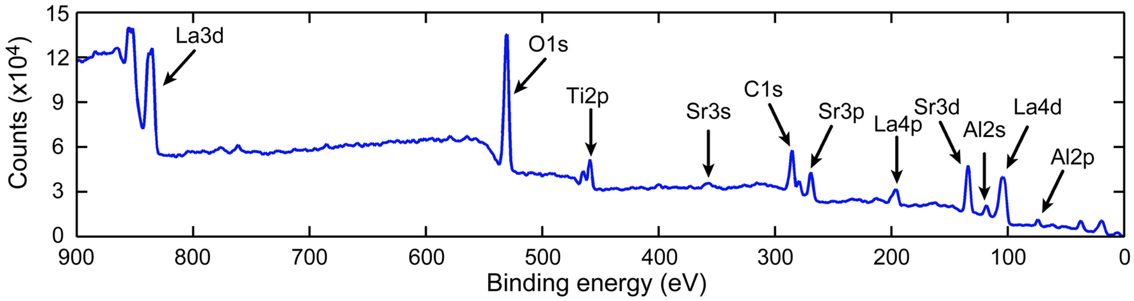

Supplementary Figure 3. X-ray photoelectron spectroscopy (XPS) survey scan of 4 uc LAO/STO with corresponding peak assignments.

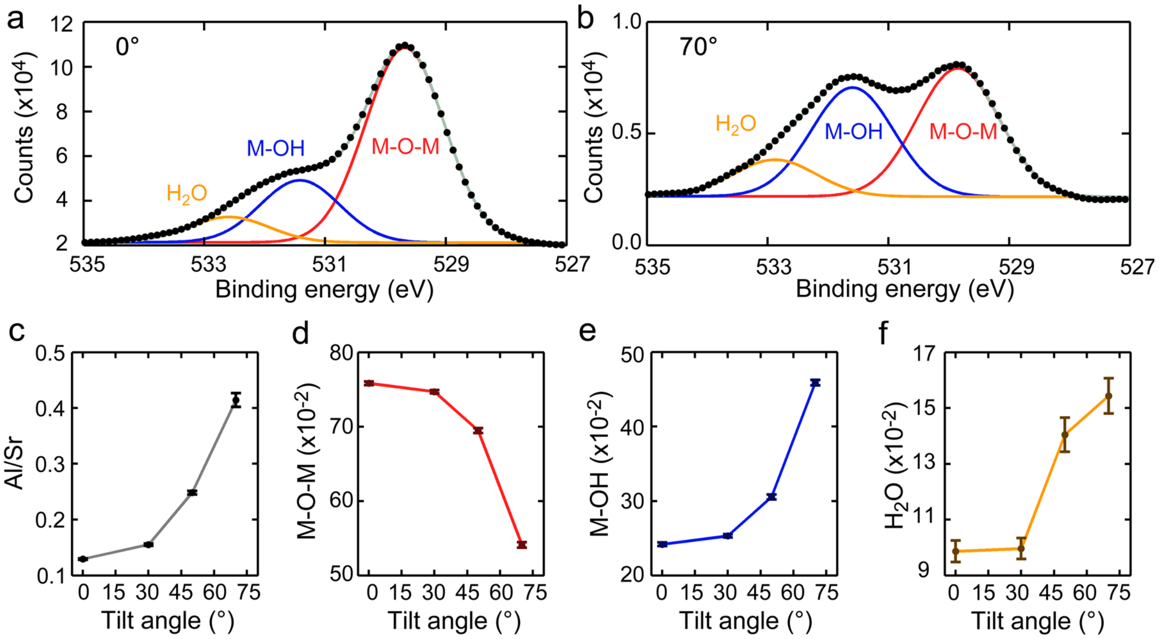

Supplementary Figure 4. Tilted XPS study of 4 uc LAO/STO to confirm peak assignments. XPS of 4 uc LAO on STO showing the O1s region at 0º tilt (**a**) and 70º tilt (**b**). Each spectrum is fit to the sum of three Gaussians assigned to free water (H_2O), protonated oxygen (M-OH), and oxygen in the perovskite lattice (M-O-M). (**c**) The Al/Sr peak area ratio increases with increasing tilt, confirming that Al is closer to the surface. As the tilt increases, the measured concentration of M-O-M (**d**) decreases, while the measured concentrations of M-OH (**e**), and H_2O (**f**) increase, thus suggesting that the M-OH and H_2O species are localized to the surface. The peak areas for (**d-f**) are normalized by the sum of M-O-M and M-OH.

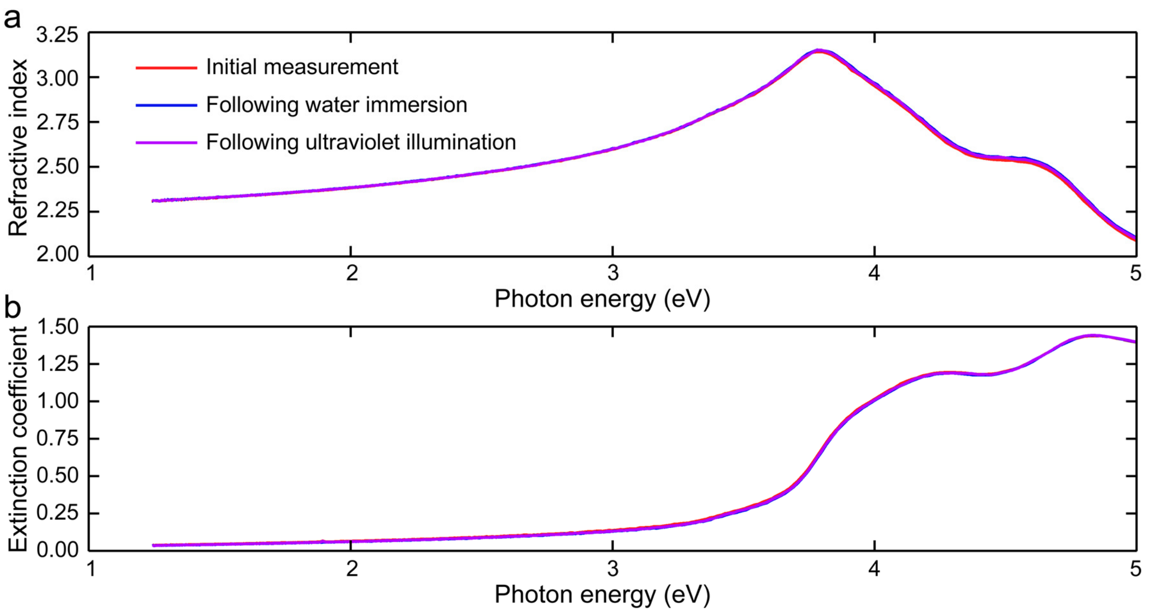

Supplementary Figure 5. Spectroscopic ellipsometry of LAO/STO interfaces reveals negligible changes of optical properties upon immersion in solvent or illumination with light. The real (**a**) and imaginary (**b**) indices of refraction were extracted by modeling the interface as a homogenous material. Care was taken to align the light source while using a 500 nm long pass filter in order to prevent extraneous exposure to UV light. Initial measurement (red), measurement following 3 min of immersion in DI water (blue), and measurement following 3 min of illumination under the light source used in Fig 1b (purple) all showed superimposable curves.

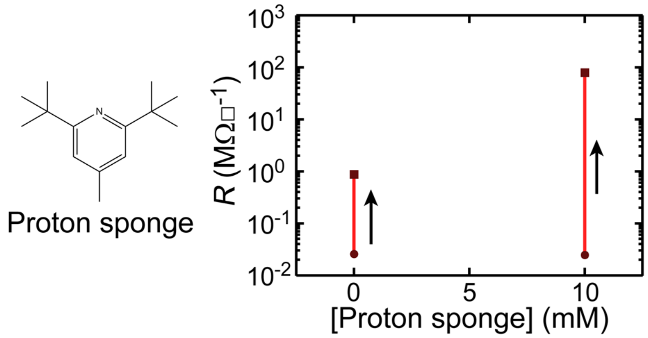

Supplementary Figure 6. *R* of 4 uc LAO/STO measured following two minute immersions in nitromethane with the “proton sponge” 2,6-di-*tert*-butyl-4-methylpyridine.

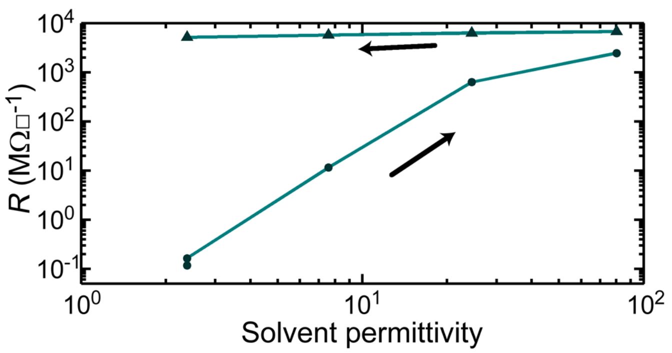

Supplementary Figure 7. *R* of 4 uc LAO/STO measured following sequential one minute immersions in solvents with different relative permittivities. From lowest to highest permittivity, the solvents were toluene, tetrahydrofuran, ethanol, and deionized water. Circles denote experiments in which the sample experienced sequentially increasing permittivity solvents and triangles denote experiments in which the sample experienced solvents with sequentially decreasing permittivity.

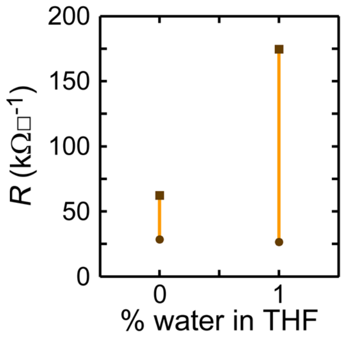

Supplementary Figure 8. *R* of 4 uc LAO/STO measured before and after two minute immersions in pure tetrahydrofuran (THF) or 1:99 DI water: THF by volume. The stark difference between these cannot be explained by a change in the permittivity; instead, this indicates that trace water could be an important determinant of the effect of non-aqueous solvent immersion.

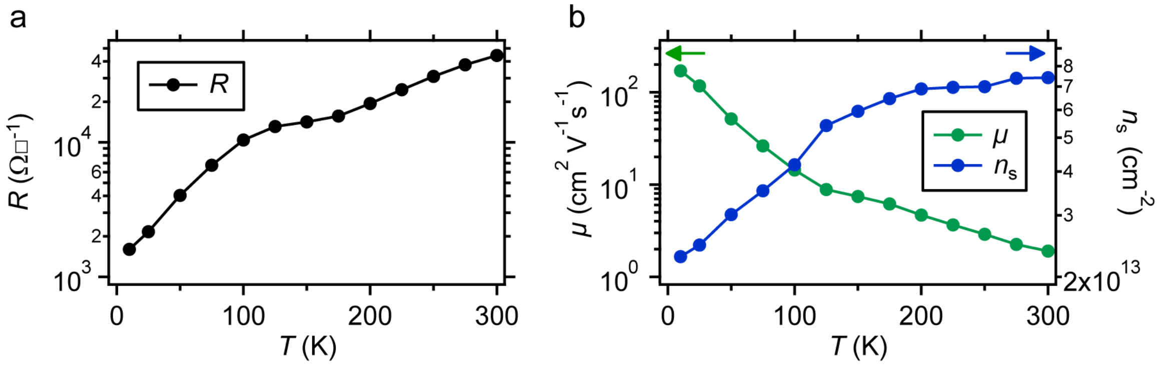

Supplementary Figure 9. (a) Temperature *T* dependence of sheet resistance *R* of a 4 uc LAO/STO sample measured after the sample was exposed to broadband light for 3 minutes. **(b)** Temperature dependence of mobility μ and carrier density n_s of a 4 uc LAO/STO sample measured after the sample was exposed to broadband light for 3 minutes.

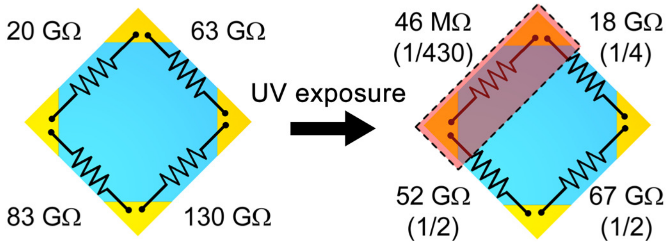

Supplementary Figure 10. Patterning LAO/STO samples using the surface-driven insulator-to-conductor transition. Two terminal resistances measured between each neighboring pad for a 4 uc LAO on STO sample following immersion in water (left) and after one edge of the sample was exposed to UV light (right). The ratios indicate the change in resistance for each measurement.

Supplementary Methods:

I. Sample preparation

Epitaxial $LaAlO_3$ (LAO) thin films were grown on $SrTiO_3$ (STO) substrates using pulsed-laser deposition (PLD) with *in situ* high-pressure reflection high-energy electron diffraction (RHEED). Low miscut (<0.10°) single-crystal STO (001) substrates were etched by buffered hydrofluoric acid for 60 seconds to obtain substrates with a B-site (TiO_2) terminated surface. Then, the substrates were annealed in a tube furnace at 1000 °C for 6 hours to make an atomically smooth surface with single unit cell height steps. For the growth of epitaxial LAO thin films, a KrF excimer laser (248 nm) beam was focused on a stoichiometric $LaAlO_3$ single crystal target and pulsed at 3 Hz frequency. The growth temperature of substrates was 550 °C and the background oxygen pressure was 10^{-3} mbar. After growing 4 unit cells (uc) of LAO with *in-situ* RHEED oscillation monitoring, the samples were slowly cooled down to room temperature.

Electrodes were patterned on samples to enable electrical characterization (Supplementary Figure 1). First, samples were spin-coated with photoresist (S1803 – The Dow Chemical Company) at 4000 rpm for 40 s with a 500 rpm s^{-1} ramping speed. The spin-coating

recipe also contained a 5 s hold at 500 rpm to spread the photoresist prior to high speed spinning. Following spin-coating, the sample was baked at 115 ºC for 75 s. Next, the sample was exposed to UV light for 7 s for an approximate dose of 150 mJ cm^{-2} at 405 nm (MA6 – Suss MicroTec AG). As these masks exposed the corners which were susceptible to edge beads, a slightly longer than standard exposure time was needed. Next, samples were developed for 60 s (MF-319 – The Dow Chemical Company) and inspected using optical microscopy to ensure developing was complete. Following photolithography, the sample was subjected to a reactive ion etch for 120 s at 13.3 Pa with 30 sccm Ar and 200 W of applied power (RIE-10NR – SAMCO Inc.). Immediately following etching, the sample was placed in an electron beam deposition system and coated with 5 nm of Cr and 45 nm of Au (PVD-75 – Kurt J. Lesker Company). The residual photoresist was then lifted off by sonicating the sample in acetone for ~5 min until the sample was visibly clean. Finally, the sample was briefly rinsed with acetone, deionized (DI) water, and isopropanol and blown dry under an N_2 stream. LAO/STO heterointerfaces were further characterized using atomic force microscopy (Dimension ICON – Bruker Corporation).

II. Electrical measurements

Van der Pauw measurements were carried out using a source meter (4200SCS – Keithley Instruments Inc.) connected to the sample which was held in a probe station (ST-500 – Janis Research Company, LLC). Unless noted otherwise, all measurements were taken in the dark under ambient atmospheric conditions. A 500 nm long pass filter (Y-50 – Edmund Optics Inc.) was used to block blue or UV light during the process of positioning the probe arms. A single Van der Pauw measurement consisted of four individual four point measurements organized such that each permutation of electrodes was tested. Each four point measurement consisted of applying bias voltages of ±200 mV while recording the source-drain current and voltages on the other two pads. These values were used to compute the sheet resistance *R*. Importantly, measurements taken while sweeping the bias voltage in 50 mV intervals between 200 mV and -200 mV revealed that, in all cases, the samples exhibited an Ohmic response.

All solvent immersion experiments were carried out in the dark or under light with the blue and UV components removed (*i.e.* cleanroom lighting). Scintillation vials were filled with ~10 mL of the solvent of interest. The sample was then placed at the bottom of the vial. Following a predetermined amount of time, the sample was removed and blown dry under flowing N_2. DI water was obtained from a filter system (Barnstead Pacific RO – Thermo Fisher Scientific Inc.).

Illumination experiments were carried out while the sample was mounted in the probe station. For broadband illumination, a mercury arc lamp (X-Cite 120Q – Excelitas Technologies Corp.) was used. At the distance at which the sample was positioned, the optical intensity in the range 400 to 500 nm was found to be 1-2 mW cm^{-2}. For monochromatic light, a monochromator (77250 series monochromator – Newport Corporation) was used to filter the light from a Xe arc lamp (66902 Series Arc Lamp – Newport Corporation).

III. X-ray photoelectron spectroscopy (XPS)

XPS measurements were performed in a commercial spectrometer (Thermo Scientific ESCALAB 250Xi) with a monochromated Al Kα radiation source and care taken to avoid exposure to visible light. The electron flood gun was turned on in order to reduce surface charging. The pressure in the vacuum chamber during the analysis was less than 10^{-9} bar. Each scan was recorded as the average of five sequential scans. Each sample was measured in three distinct areas to establish statistical error bars for the measurements. After scanning, all the

binding energies (BEs) were referenced to the adsorbed carbon C1s peak, which was set to 284.8 eV. Fitting was performed via an automated routine implemented in MATLAB wherein the bands were fit to a sum of Gaussian peaks plus a linear background. For example, the O1s region was fit to the sum of three Gaussians. This number was chosen because this is the maximum number that appreciably affected the mean square error. The peak widths were constrained to be the same for all peaks in a given scan and were found to be 1.02 ± 0.05 eV for all measurements.

XPS peaks were assigned by considering the result of the tilting experiment (Supplementary Figure 4) and literature values for the expected binding energies of oxygen species. In the O1s region, three peaks were identified centered on 529.7, 531.6, and 532.6 eV. The peak centered on 529.7 eV is attributed to M-O-M oxygen in both STO and LAO, in agreement with ranges from the literature of 529.2 to 529.4 eV for STO[1,2] and 529.2 to 529.6 eV for LAO.[3,4] The peak centered on 531.6 eV was attributed to surface hydroxylate (*i.e.* M-OH) in agreement with literature values for hydroxylated species on alumina being located at 531.5 eV[5] and reports that hydroxylated peak in LAO should be higher in energy than the M-O-M peak by 1.6 eV.[6] Finally, the peak centered on 532.6 eV was attributed to adsorbed water based on the observation that adsorbed water is expected to result in a peak 3 eV higher in energy than the M-O-M peak in LAO.[6] These assignments are consistent with the observation from the tilting experiment (Supplementary Figure 4) that the M-OH and H_2O species are closer to the surface than the M-O-M species, which should be distributed throughout the material.

IV. Optical characterization

The optical properties of 4 uc LAO/STO samples were characterized using spectroscopic ellipsometery (M-2000 - J.A. Woollam Co. Inc.) measured at 55º, 65º, and 75º from normal incidence. The resulting angles were used to compute a real and imaginary refractive index which are shown in Supplementary Figure 5. Note that there was no appreciable change following immersion in water or exposure to light. Alignment was performed through a 500 nm long pass filter to prevent triggering the light-driven transition.

V. Solvent immersion

All solvent immersion experiments were carried out in the dark or under light with the blue and UV components removed (*i.e.* cleanroom lighting). Scintillation vials were filled with ~10 mL of the solvent of interest. Anhydrous solvents were used to mitigate trace water contamination and were used within 10 min of introduction to air. For solvents not available in a dry state, 50 mL volumes were stored for two days in the presence of a molecular sieve. The sample was then placed at the bottom of the vial. Following a predetermined amount of time, the sample was removed and blown dry under flowing N_2. DI water was obtained from a filter system (Barnstead Pacific RO – Thermo Fisher Scientific Inc.). Acidic or salt solutions were prepared by introducing known quantities of sulfuric acid or sodium sulfate. All other solvents (*i.e.* acetone, ethanol, isopropanol, toluene, and tetrahydrofuran) were obtained from Sigma-Aldrich Company, LLC.

VI. Temperature-Dependent Transport

In order to further explore the light induced conductivity, a 4 uc LAO/STO sample was exposed to broadband light for 3 minutes and subsequently loaded into a Physical Properties Measurement System (PPMS – Quantum Design). Van der Pauw and magnetotransport

measurements were carried out in the temperature range 10 K to 300 K to determine the sheet resistance *R*, carrier density n_s, and mobility μ. Data is shown in Supplementary Figure 9. Samples in the insulating state were too resistive to accurately determine n_s or μ.

VII. Patterning experiments

In order to test the ability to pattern the conductive state, samples in the insulating state were exposed to light using a mask aligner (MA6 – Suss MicroTec AG) for 1 s resulting in ~25 mW cm^{-2} at 405 nm. Data is shown in Supplementary Figure 10.

Supplementary References:

1 Vasquez, R. P. $SrTiO_3$ by XPS. *Surface Science Spectra* **1**, 129-135, (1992).

2 Das, S., Liu, D., Janardhanam, V., Choi, C.-J. & Hahn, Y.-B. Stoichiometry-controlled growth of $Ba_xSr_{1-x}TiO_3$ thin films and their electrical behavior in heterojunction assemblies. *RSC Advances* **2**, 10255-10261, (2012).

3 Vasquez, R. P. $LaAlO_3$(100) by XPS. *Surface Science Spectra* **1**, 361-366, (1992).

4 Haack, L. P., deVries, J. E., Otto, K. & Chattha, M. S. Characterization of lanthanum-modified γ-alumina by X-ray photoelectron spectroscopy and carbon dioxide absorption. *Applied Catalysis A: General* **82**, 199-214, (1992).

5 Lefèvre, G., Duc, M., Lepeut, P., Caplain, R. & Fédoroff, M. Hydration of γ-Alumina in Water and Its Effects on Surface Reactivity. *Langmuir* **18**, 7530-7537, (2002).

6 Kienzle, D., Koirala, P. & Marks, L. D. Lanthanum aluminate (110) 3 x 1 surface reconstruction. *Surf. Sci.* **633**, 60-67, (2015).
